# Gastrointestinal carriage of Klebsiella pneumoniae in a general adult population: a cross-sectional study of risk factors and bacterial genomic diversity

**DOI:** 10.1080/19490976.2021.1939599

**Published:** 2021-06-28

**Authors:** Niclas Raffelsberger, Marit Andrea Klokkhammer Hetland, Kristian Svendsen, Lars Småbrekke, Iren Høyland Löhr, Lotte Leonore Eivindsdatter Andreassen, Sylvain Brisse, Kathryn E. Holt, Arnfinn Sundsfjord, Ørjan Samuelsen, Kirsten Gravningen

**Affiliations:** a Department of Microbiology and Infection Control, University Hospital of North Norway, Tromsø, Norway; b Department of Medical Biology, Faculty of Health Sciences, UiT the Arctic University of Norway, Tromsø, Norway; c Department of Medical Microbiology, Stavanger University Hospital, Stavanger, Norway; d Department of Biological Sciences, Faculty of Mathematics and Natural Sciences, University of Bergen, Bergen, Norway; e Department of Pharmacy, Faculty of Health Sciences, UiT the Arctic University of Norway, Tromsø, Norway; f Institut Pasteur, Biodiversity and Epidemiology of Bacterial Pathogens, Paris, France; g Department of Infectious Diseases, Central Clinical School, Monash University, Melbourne, Australia; h Department of Infection Biology, Faculty of Infectious and Tropical Diseases, London School of Hygiene & Tropical Medicine, London, UK; i Norwegian National Advisory Unit on Detection of Antimicrobial Resistance, Department of Microbiology and Infection Control, University Hospital of North Norway, Tromsø, Norway; j Department of Infection Control and Preparedness, Norwegian Institute of Public Health, Oslo, Norway

**Keywords:** *Klebsiella pneumoniae*, carriage, risk factors, general population, bacterial genomics

## Abstract

Antibiotic resistant *Klebsiella pneumoniae* is a leading public health threat and gastrointestinal carriage is an established risk factor for subsequent infections during hospitalization. Our study contributes new knowledge of risk factors for gastrointestinal carriage and the genomic population structure of *K. pneumoniae* colonizing humans in a representative sample of a general population in a community setting. Altogether, 2,975 participants (54% women) >40 y in the population-based Tromsø Study: Tromsø7, Norway (2015–2016) were included. Fecal samples were screened for *K. pneumoniae*, which were characterized using whole-genome sequencing. Risk factors for carriage were analyzed using multivariable logistic regression on data from questionnaires and the Norwegian Prescription Database. Prevalence of *K. pneumoniae* gastrointestinal carriage was 16.3% (95% CI 15.0–17.7, no gender difference). Risk factors associated with carriage included age ≥60 y, travel to Greece or Asia past 12 months (adjusted odds ratio 1.49, 95% CI 1.11–2.00), Crohn’s disease/ulcerative colitis (2.26, 1.20–4.27), use of proton pump inhibitors (1.62, 1.18–2.22) and non-steroidal anti-inflammatory drugs past 6 months (1.38, 1.04–1.84), and antibiotic use the last month (1.73, 1.05–2.86). Prevalence was higher among those having used combinations of drug classes and decreased over time with respect to preceding antibiotic use. The *K. pneumoniae* population was diverse with 300 sequence types among 484 isolates distributed across four phylogroups. Only 5.2% of isolates harbored acquired resistance and 11.6% had virulence factors. Identification of risk factors for gastrointestinal carriage allows for identification of individuals that may have higher risk of extraintestinal infection during hospitalization. The findings that specific diseases and drugs used were associated with carriage show an impact of these possibly through modulating the human gut microbiota promoting colonization. The diverse population structure of carriage isolates reflects the ecologically adaptive capacity of the bacterium and challenges for vaccine prospects and the identification of reservoirs as a potential source for human colonization.

## Introduction

*Klebsiella pneumoniae* (Kp) is a key pathogen associated with nosocomial infections frequently accompanied by antibiotic resistance.^1,2^ As an opportunistic pathogen, Kp is particularly problematic among neonates, elderly, immunocompromised, and patients with underlying chronic diseases, and commonly causes pneumonia, urinary tract infections, and bacteremia.^1^ Additionally, the problem is exacerbated by the emergence and spread of community-acquired hypervirulent Kp causing infections in healthy individuals, usually presenting as pyogenic liver abscess occasionally accompanied with metastatic spread, but also as meningitis or endophthalmitis.^3^

Kp is known for its high prevalence and diversity of antibiotic resistance genes that challenge treatment options due to infections with multi-drug resistant variants. In Europe, antibiotic resistant Kp was responsible for more than 89,000 infections and 5,600 attributable deaths in 2015.^4^ Several new antibiotic resistance genes were first discovered within Kp before spreading to other pathogens.^5^ Consequently, The World Health Organization considers antibiotic-resistant Kp as a critical-priority bacterium in antibiotic research and development.^6^

Recent taxonomic updates show that Kp subdivides into five different species comprising seven phylogroups (Kp1-Kp7) referred to as the *K. pneumoniae* species complex (KpSC).^7,8^ The phylogroups include *K. pneumoniae sensu stricto* (Kp1), *K. quasipneumoniae* subsp. *quasipneumoniae* (Kp2), *K. variicola* subsp. *variicola* (Kp3), *K. quasipneumoniae* subsp. *similipneumoniae* (Kp4), *K. variicola* subsp. *tropica* (Kp5), *‘K. quasivariicola’* (Kp6) and *K. africana* (Kp7).^7–12^ Herein, we refer to “Kp” for all members of the KpSC unless otherwise specified. Kp has a broad environmental distribution, and transmission routes to humans are currently not well defined.^8^

Gastrointestinal carriage of Kp as a reservoir for healthcare-associated Kp infections was established in the early 1970s.^13^ Recent genomic studies show that gastrointestinal carriage is a risk factor for subsequent extraintestinal infections, and ~50% of Kp bloodstream infections are caused by the patient’s own gut isolates.^14,15^ Moreover, the relative abundance of Kp in the gastrointestinal tract is associated with an increased risk of Kp bacteremia.^16^

Cross-sectional studies have shown that Kp gastrointestinal carriage prevalence varies from 6% to 88% depending on geographical locations, detection methods and the populations investigated.^14,15,17–19^ However, we have a sparse understanding of risk factors for Kp gastrointestinal carriage and the population structure of Kp in the general human population. In a recent cross-sectional study of 911 pregnant women in low-income countries, Huynh *et al*. identified various country-specific environmental exposure factors linked to Kp gastrointestinal carriage and a diverse Kp population structure.^19^

Here, we investigated the prevalence of Kp carriage and associated risk factors among 2,975 participants in a cross-sectional study of a representative sample of a general adult population in Norway, a country with a low prevalence of antibiotic resistance and restricted antibiotic use. Additionally, we elucidated the Kp genomic population structure of carriage isolates.

## Results

We analyzed data from 2,975 participants (1,615 women, 54.3%, Suppl. Table 1) 40 y and older. Median age of the participants was 65.0 y (Interquartile Range 59–71 y, no gender difference). Altogether, we identified 484 Kp carriers corresponding to a prevalence of 16.2% (95% CI 14.5–18.1) among women and 16.3% (14.4–18.4) among men.

### Kp gastrointestinal carriage and associated factors

In analysis adjusting for all of the explanatory variables (Suppl. Figure 1), Kp gastrointestinal carriage was associated with age 60 y and older (compared to the reference group 40–49 y), self-reported travel to Greece or Asia during the preceding 12 months (AOR 1.49, 1.11–2.00) and Crohn’s disease/ulcerative colitis (2.26, 1.20–4.27) (Table 1). Furthermore, carriage was associated with the use of proton pump inhibitors (PPIs) (1.62, 1.18–2.22) and non-steroidal anti-inflammatory drugs (NSAIDs) (1.38, 1.04–1.84) in the past 6 months, and antibiotic use in the last month (1.73, 1.05–2.86).

**Table 1.** *K. pneumoniae* (Kp) gastrointestinal carriage and associated factors among 2,975 participants in the Tromsø Study: Tromsø7 in crude and multivariable logistic regression analyses

	% (Kp)	*n* (Kp)	*N*	OR	95% CI	*p*-Value	AOR	95% CI	*p*-Value
**Age** (y)						0.024			0.128
40–49	10.8	37	344	1.00			1.00		
50–59	15.4	67	435	1.51	0.98–2.32		1.34	0.85–2.11	
60–69	17.3	222	1,286	1.73	1.20–2.51		1.56	1.06–2.30	
70–84	17.4	158	910	1.74	1.19–2.55		1.56	1.03–2.36	
**Current daily smoking**						0.798			0.717
No	16.2	421	2,598	1.00			1.00		
Yes	15.7	55	351	0.96	0.71–1.31		0.94	0.66–1.34	
**Alcohol consumption frequency**						0.021			0.050
Never to < monthly	16.4	164	998	1.00			1.00		
2–4/month to 2–3/week	16.9	298	1,765	1.03	0.84–1.27		1.13	0.88–1.46	
≥4/week	9.1	18	198	0.51	0.31–0.85		0.61	0.35–1.05	
**Alcohol units/occasion**						0.501			0.365
1–4	16.2	412	2,540	1.00			1.00		
≥5	14.1	20	142	0.85	0.52–1.37		0.79	0.47–1.32	
**Travel abroad past 12 months^a^**						0.024			0.009
No	15.5	196	1,276	1.00			1.00		
Greece or Asia	20.3	102	502	1.39	1.07–1.82		1.49	1.11–2.00	
All other countries	15.3	179	1,171	0.99	0.79–1.23		0.97	0.76–1.26	
**Hospitalization past 12 months**						0.145			0.844
No	15.9	412	2,588	1.00			1.00		
Yes	19.0	67	353	1.24	0.93–1.65		1.03	0.74–1.44	
**Diabetes mellitus^b^**						0.011			0.056
No	16.0	427	2,674	1.00			1.00		
Yes	23.5	40	170	1.62	1.12–2.34		1.82	0.99–3.36	
**Crohn’s disease/ulcerative colitis**						0.012			0.012
No	16.0	452	2,831	1.00			1.00		
Yes	28.3	17	60	2.08	1.18–3.68		2.26	1.20–4.27	
**Proton pump inhibitors past 6 months^c^**						<0.001			0.003
No	15.3	404	2,632	1.00			1.00		
Yes	23.3	80	343	1.68	1.28–2.20		1.62	1.18–2.22	
**NSAIDs past 6 months^d^**						0.016			0.028
No	15.6	397	2,545	1.00			1.00		
Yes	20.2	87	430	1.37	1.06–1.78		1.38	1.04–1.84	
**Antibiotic systemic use past 1 month^e^**						0.001			0.032
No	15.8	453	2,866	1.00			1.00		
Yes	28.4	31	109	2.12	1.38–3.25		1.73	1.05–2.86	
**Metformin past 6 months^f^**						0.090			0.764
No	16.0	460	2,867	1.00			1.00		
Yes	22.2	24	108	1.50	0.94–2.38		0.89	0.40–1.95	
**Thyroid hormones past 6 months^g^**						0.065			0.333
No	15.9	431	2,714	1.00			1.00		
Yes	20.3	53	261	1.35	0.98–1.86		1.20	0.83–1.75	

N, denominator; OR, odds ratio; CI, confidence interval; AOR, adjusted odds ratio; NSAIDs, non-steroidal anti-inflammatory drugs. AOR adjusted for age, current daily smoking, alcohol consumption frequency, alcohol units/occasion, travel abroad past 12 months, hospitalization past 12 months, diabetes mellitus, Crohn’s disease/ulcerative colitis and drug use according to the Norwegian Prescription Database (A02BC, M01, J01, A07AA09, P01AB01, A10BA02, H03AA). The multivariable model contains 2,446 participants with complete information on all variables. ^a^Traveled outside the Nordic countries >1 week duration in the past 12 months. ^b^20 participants who answered “Yes, previously” were excluded. ^c^A02BC, drugs used for peptic ulcer and gastro-esophageal reflux disease. **^d^**M01, anti-inflammatory and anti-rheumatic products (non-steroids), anti-inflammatory/anti-rheumatic agents in combination and specific anti-rheumatic agents. ^e^J01, A07AA09, P01AB01, antibacterials for systemic use, intestinal antiinfectives and nitroimidazole derivates used as antiprotozoals (metronidazole). ^f^A10BA02, blood glucose lowering drug used in diabetes. ^g^H03AA, natural and synthetic thyroid hormones.

Analysis of the significant variables in the multivariable model for the three most frequent species (Kp1, Kp2 and Kp3) is presented in Suppl. Table 2. In this descriptive analysis, we compared participants colonized by a single phylogroup to Kp non-carriers, excluding carriers of any other phylogroup. Kp1 was the dominating phylogroup responsible for the associations to selected variables, except for NSAID use which is solely significantly associated with Kp2. Kp3 is associated with PPI and antibiotic use.

**Figure 1.**
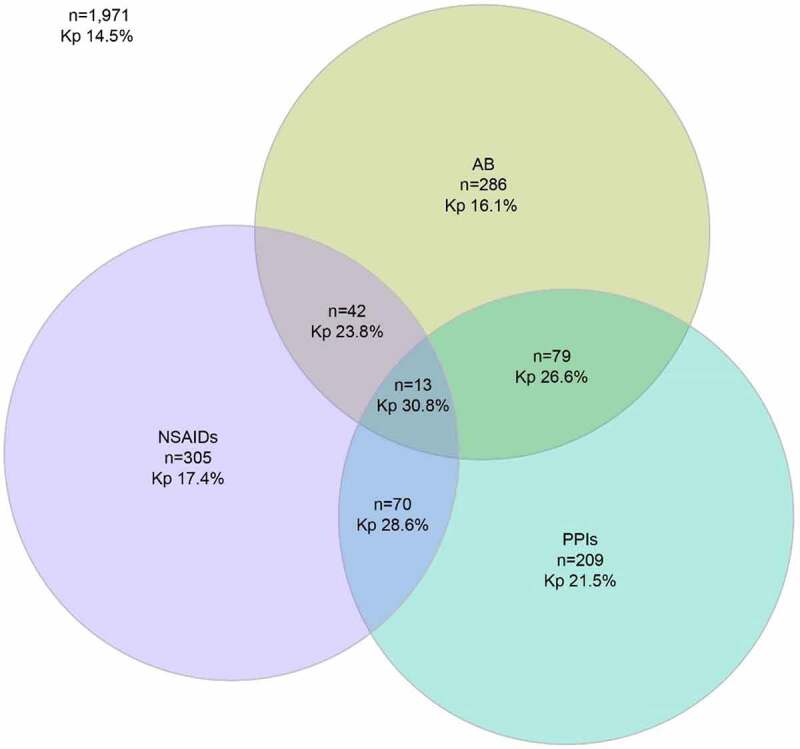
Proportional Venn diagram of Kp carriage prevalence related to statistically significantly associated drug classes (antibiotics (AB), nonsteroidal anti-inflammatory drugs (NSAIDs) and proton pump inhibitors (PPIs)) in the past 6 months among 2,975 study participants

The Kp prevalence of the three statistically significantly associated drug classes (usage in past 6 months) in relation to Kp carriage is shown in Figure 1. Kp prevalence was 16.1% among 286 antibiotic-only users, 17.4% among 305 NSAID-only users, and 21.5% among 209 PPI-only users. Kp prevalence increased in each overlapping area for two drug classes, and further increased to 30.8% in the overlapping area for all three.

**Figure 2.**
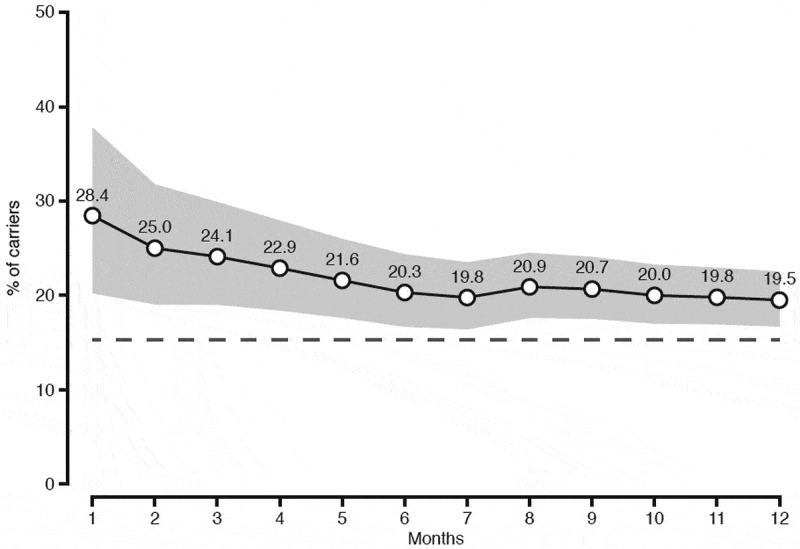
Cumulative change in the proportion of Kp carriers among those who had used antibiotics 1–12 months before the fecal sampling. Shaded gray area represents the 95% CI. The time period at each specified month includes data for the preceding months. The dashed black line indicates the prevalence of carriage in the non-antibiotic using population (15.2%)

Looking at the cumulative change in proportion of Kp carriers associated with antibiotic use during 1–12 months before fecal sampling, we found that Kp carriage prevalence was highest among those with antibiotic use in the past month (28.4%) and past 2 months (25.0%), decreasing to around 20.0% in the past 6–12 months (Figure 2). In contrast, prevalence of Kp carriage was significantly lower (15.2%) in the non-antibiotic using population.

### Kp phylogeny and diversity

**Figure 3.**
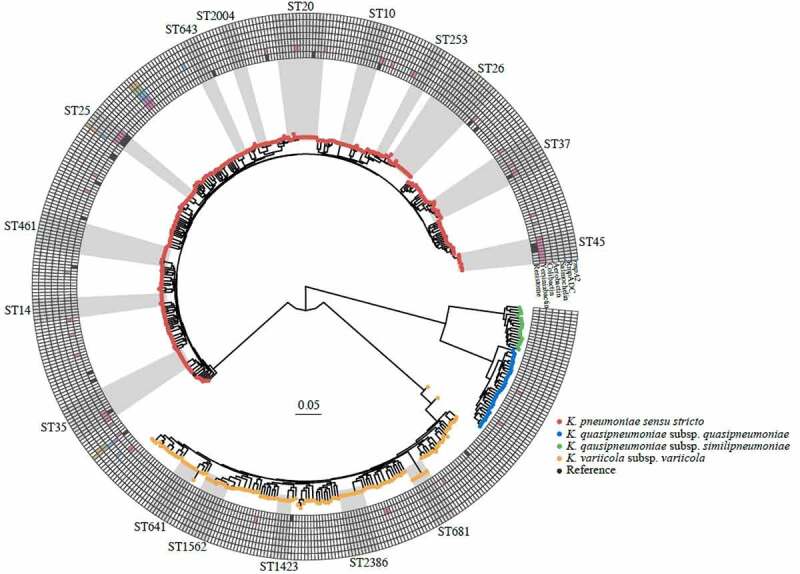
Core chromosomal maximum likelihood phylogeny of the 484 Kp genomes. The tips are colored by species. The heatmap shows presence (color) or absence (white) of acquired resistance genes (innermost ring) or virulence factors (remaining six rings). Clades corresponding to STs with five or more genomes are highlighted and labeled

Phylogenetic analysis based on whole-genome sequencing of 484 *K. pneumoniae* isolates identified three species distributed in four phylogroups with a predominance of *K. pneumoniae sensu stricto* (Kp1, 62.6%), followed by *K. variicola* subsp. *variicola* (Kp3, 27.7%), *K. quasipneumoniae* subsp. *quasipneumoniae* (Kp2, 6.4%), and *K. quasipneumoniae* subsp. *similipneumoniae* (Kp4, 3.3%) (Figure 3, Suppl. Table 3). Phylogroups Kp5-Kp7 were not identified. Using multilocus sequence typing (MLST), we found a high degree of genotypic diversity (Simpson diversity index 99.5%) with a total of 300 different STs, including 96 (32%) novel STs (Suppl. Figure 2).^20^ The majority (79%) of the STs were represented by a single isolate. Only 17 STs (5.7%) were represented by more than five isolates. The most frequent were ST20 (n = 15, 3.1%), ST26 (n = 13, 2.7%), ST35 (n = 9, 1.9%), ST37 (n = 9, 1.9%), and ST2386 (n = 9, 1.9%). With regard to clonal relatedness, we found seven ST35 and three ST25 isolates with a range of zero to five SNPs (Suppl. Table 4), and seven to eight SNPs (Suppl. Table 5), respectively, indicating a recent clonal spread. No close clonal relatedness was identified among the remaining frequent STs (Suppl. Table 6).

### Antimicrobial resistance and plasmid content

The prevalence of resistance was low and none of the isolates were resistant to cefotaxime, meropenem, aztreonam, or ciprofloxacin (Suppl. Figure 3). Resistance was observed against amoxicillin-clavulanic acid (0.6%), ceftazidime (0.2%), gentamicin (0.4%), cefuroxime (0.6%), trimethoprim-sulfamethoxazole (1.7%), piperacillin-tazobactam (4.8%), and mecillinam (5.0%). This was in concordance with the low number of intrinsic and acquired resistance genes (Figure 3, Suppl. Table 3). A high sequence diversity of species-specific intrinsic narrow-spectrum chromosomal β-lactamase gene alleles were identified (Suppl. Figures 4, 5, 6, and 7, Suppl. Table 3). Three ampicillin-susceptible isolates harbored either deleted *bla*_SHV_/*bla*_LEN_ genes or a premature stop codon in *bla*_SHV_. Only 5.2% of the isolates harbored an acquired resistance gene (median number: 2, Suppl. Table 3). *Bla*_SHV-51_ and *bla*_SHV-52_ were assigned by Kleborate as extended-spectrum β-lactamase and inhibitor-resistant β-lactamase, respectively. However, both isolates were phenotypically susceptible to all tested β-lactams and β-lactam-inhibitor combinations. Thirty-two plasmid replicon types were identified among 393/484 (81.2%) isolates with IncFIB(K) (25.2%), Col (pHAD28) (15.0%), IncFIB(K) (pCAV1099-114) (11.4%), IncFIA (HI1) (10.5%), and IncFII (pKP91) (9.8%) the most frequent (Suppl. Table 3). The median number of replicon types per isolate was two.

### Virulence factors and serotype prediction

The majority of isolates (n = 428, 88.4%) did not contain known acquired virulence determinants (Suppl. Table 3). Five *K. pneumoniae sensu stricto* isolates (1.0%) of different STs were defined as hypervirulent, including one isolate of the known hypervirulent ST23 clone.^3^ These five isolates harbored aerobactin (*iuc1* or *iuc2*), salmochelin (*iro1* or *iro2*) and *rmpA* (Suppl. Table 3); three additionally carried the yersiniabactin (*ybt*) locus. Three of the hypervirulent isolates harbored the capsular synthesis locus (KL) 1 or KL2, previously described as hypervirulence associated;^3^ the other two harbored KL23. Overall, the siderophore loci *ybt, iuc*, and *iro* were present in 10.7% (n = 52), 1.4% (n = 7), and 1.0% (n = 5) of the isolates, respectively (Suppl. Table 3). A total of 96 defined KL types were found (Suppl. Figure 8), with KL10 being the most frequent (n = 21; 4.7%) followed by KL28 (n = 18; 4.0%) and KL22 (n = 17; 3.8%). KL2 accounted for 2.9% (n = 13) and KL1 for only 0.9% (n = 4). We observed 11 different defined O-antigen types in 477 isolates (Suppl. Figure 9) with O1 (n = 133; 27.9%) being the most frequent followed by O3/3a (n = 113; 23.7%), O2 (n = 76; 15.9%), O5 (n = 58; 12.2%), and O3b (n = 47; 9.9%), accounting for 90% of the isolates. Five isolates harbored an O-locus with an unknown O-type.

## Discussion

In this study of a large representative sample of a general population aged 40 y and older, we detected an overall Kp gastrointestinal carriage prevalence of 16.3% with no gender difference. We found that Kp carriage was associated with age 60 y and older, reported Crohn’s disease/ulcerative colitis, travel to Asia and Greece in the preceding 12 months, and recent use of PPIs, NSAIDs, and antibiotics. We showed that the Kp population among adults in the community setting in a high-income country with low antibiotic use was highly diverse and characterized by a low prevalence of acquired antibiotic resistance and virulence determinants.

The identified Kp carriage prevalence of 16.3% is lower than that observed among pregnant women in low-income countries (40–66%)^19^ and among healthy adults in Asian countries (19–88%),^18^ but in range with a hospital admission study in Australia (6–19%)^14^ and hospitalized patients in the USA (23%).^15^ This could be explained by the different populations investigated (e.g. healthy individuals, hospitalized patient groups, geographic setting, and/or ethnicity), but also by the sampling and detection strategies. Consistent with the culture-based Australian study of hospitalized patients on admission, we found that Kp carrier prevalence increased with age.^14^ The association of Kp gastrointestinal carriage with travel abroad, irrespective of resistance phenotype, has to our knowledge not been demonstrated before. The higher Kp prevalence associated with travel to Asia could be explained by the observed high carriage among people in this region.^17–19^

The increased prevalence of Kp carriage associated with Crohn’s disease/ulcerative colitis could be due to disease-specific gut microbiota alterations. The gut microbiota of patients with Crohn’s disease departs from the normal state as microbial diversity is significantly diminished including an increased abundance of *Enterobacterales*.^21^ A review by Kaur *et al*. emphasizes the possible role of Kp in the pathogenesis of lower intestinal tract diseases.^22^ This corresponds to our findings of a higher Kp carrier prevalence among participants with self-reported Crohn’s disease/ulcerative colitis. However, the pathogenesis of Crohn’s disease/ulcerative colitis is complex and involves an interplay between different factors which may include the microbiota composition.^23^

A large number of non-antibiotic drugs have been shown to inhibit growth of one or more representative bacterial species in the human gastrointestinal tract.^24^ The positive association of PPI and/or NSAID use with Kp carriage further underpins the influence of non-antibiotic drugs on fecal microbiota and the selection of specific bacterial species. PPI use is implicated in altered gut microbiota composition, bacterial colonization patterns, including multi-drug resistant microorganisms, and increased susceptibility to enteric bacterial infections.^25–28^ Although no direct evidence exists, it may be possible that Kp as a major etiological agent of liver abscess,^29^ particularly in Asian countries, could be linked to the observation that PPI therapy is associated with an increased risk of cryptogenic liver abscess.^30^ The role of NSAIDs as a risk factor is unclear, but NSAID use has been shown to influence the gastrointestinal microbiota toward a higher relative abundance of *Enterobacteriaceae*.^31^ Interestingly, in the phylogroup subanalysis, we found NSAID users solely significantly associated with Kp2, compared to the other significant variables which were dominantly associated with Kp1 (Suppl. Table 2). However, the data must be interpreted with caution due to the low number of cases in some subgroups.

Systemic antibiotics more obviously influence the microbiota composition. Kp is intrinsically resistant to aminopenicillins and carboxypenicillins, and thus has a selective advantage compared to other bacteria during treatment with penicillins, which constitutes approximately half of human antibiotic use in Norway.^32^ Antibiotics leave an imprint on the gastrointestinal bacterial community after treatment is removed, ranging from weeks to years in different studies.^33^ This is consistent with our finding of a significant increase in Kp prevalence related to antibiotic exposure even 12 months prior to fecal sampling. Notably, our data display a quantitative time–response relationship between antibiotic use and Kp carriage. The prevalence of Kp carriage decreased among individuals sampled from one to six-month post-exposure and reached a plateau at 5% above that of the non-antibiotic using population sampled at 6–12 months post-exposure. The latter indicates a potentially long-lasting effect on fecal Kp occurrence after antibiotic exposure.

The findings that specific diseases and drug treatments, often used in hospital settings, were associated with Kp gastrointestinal carriage merit further research to understand how these modulate the gastrointestinal tract microbiota promoting Kp colonization. Diseases,^21^ drug use,^25,31^ and diet profiles^34^ which are shown to be associated with an increased abundance of *Proteobacteria* may consequently also be associated with Kp carriage. In contrast, microbiome compositions favoring *Firmicutes* and *Bacteroidetes* were associated with a lower risk of Gram-negative intestinal domination and corresponding bloodstream infections.^35^ Further studies are required to investigate the potential relationship between microbial composition and Kp carriage.

Alcohol consumption is an established factor in altering the gastrointestinal microbiota,^36^ however, the effect of alcohol on the abundance of different taxonomic phyla is vague.^37^ Studies investigating oropharyngeal Kp colonization have found a higher prevalence among alcoholic patients compared to controls.^38,39^ In contrast, our data may indicate that more frequent alcohol intake might be associated with lower Kp gastrointestinal prevalence, but further studies including more cases are required.

Comparable to previous reports on Kp carriage isolates in pregnant women in the community-setting in low-income countries^19^ and hospitalized patients in high-income countries,^14,15^ we found a phylogenetically highly diverse Kp population with dominance of *K. pneumoniae sensu stricto*. This indicates individually adapted Kp populations with limited interconnection. The observed diversity in our study may also be an underestimation due to the selection of only one colony for sequencing. Considering the evidence that a high proportion of Kp extraintestinal infections originate from the patients’ own carriage isolates,^14,15^ we assume that the Kp carriage population structure among individuals in the community is partly mirrored in the clinical setting. This is in line with the finding that six of the ten most prevalent STs of the carriage isolates also are among the ten most frequently observed STs in an ongoing Kp bacteremia study in Norway (unpublished data; Fostervold *et al*.). The high bacterial diversity provides important data in terms of vaccine prospects and the identification of reservoirs as a potential source for human Kp colonization.^5,40^ The low level of antibiotic resistance in our study is consistent with national data in the clinical setting and reflects the relatively low antibiotic consumption in Norway.^32^

This is the first study investigating the prevalence of Kp gastrointestinal carriage and associated risk factors in a large representative sample of a general adult population in a community setting in a high-income country. An important methodological strength is the high study attendance rate. We used data from nearly 3,000 people aged >40 y, thereby avoiding the substantial selection bias related to convenience samples in healthcare settings. Major strengths also include the combined use of questionnaire data, drug use data from a national registry database, and both phenotypic and genomic laboratory results. The high-quality drug data enabled us to detect increasing Kp prevalence among participants having used two or three drug classes simultaneously, and additionally to assess the cumulative change over time in proportion of Kp carriers associated with antibiotic use.

The age restriction is a limitation as we only studied adults >40 y. It would have been interesting to analyze Kp prevalence among those younger than 40 whom use less antibiotics and have fewer chronic diseases.^32^ As we found that Kp prevalence increased with age, extrapolation may suggest younger adults to have a lower prevalence. Diet and oral hygiene are well-known factors effecting gastrointestinal microbial composition^34,41^; however, such data were not available in our study. Due to lack of resources, we did not screen all available fecal samples for Kp carriage which would have further increased the precision of the estimates. We used selective SCAI medium for detection of Kp, as this strategy has been shown to have a high Kp recovery supporting the growth of all Kp phylogroups.^42^ However, we acknowledge that positive culturing could reflect those with a relative high abundance of Kp and that molecular-based approaches, which are less dependent on abundance and phenotypic differentiation, may detect an even higher prevalence of Kp carriage. We are currently in a follow-up study performing whole metagenomic sequencing on a subset of the fecal samples and SCAI sweeps to investigate the abundance of Kp, microbial composition of carriers and non-carriers, and the phylogroup/ST diversity within single individuals. Home sampling, as conducted in our study, could be another biasing factor. However, we controlled for this by ensuring validity of the samples by assessing bacterial growth consistent with fecal flora on nonselective media and mean transport time from sampling to laboratory arrival was only 1.8 d.

In conclusion, our findings illustrate the association of non-antibiotic drugs and inflammatory bowel diseases with increased prevalence of Kp gastrointestinal carriage that warrants considerations with regard to risk stratification in the prevention of healthcare-associated infections and opens up for potential future gut microbiome modulation interventions. The highly diverse population structure of Kp colonizing humans illustrates the capacity for adaptive diversification of this species complex. This is challenging for vaccine prospects and complicates the identification of potential Kp cross-niche transmission, which will be important in detection of animal or environmental Kp reservoirs for clinically relevant human Kp carriage and infection from a One Health perspective.

## Subjects and methods

### Study population and design

**Figure 4.**
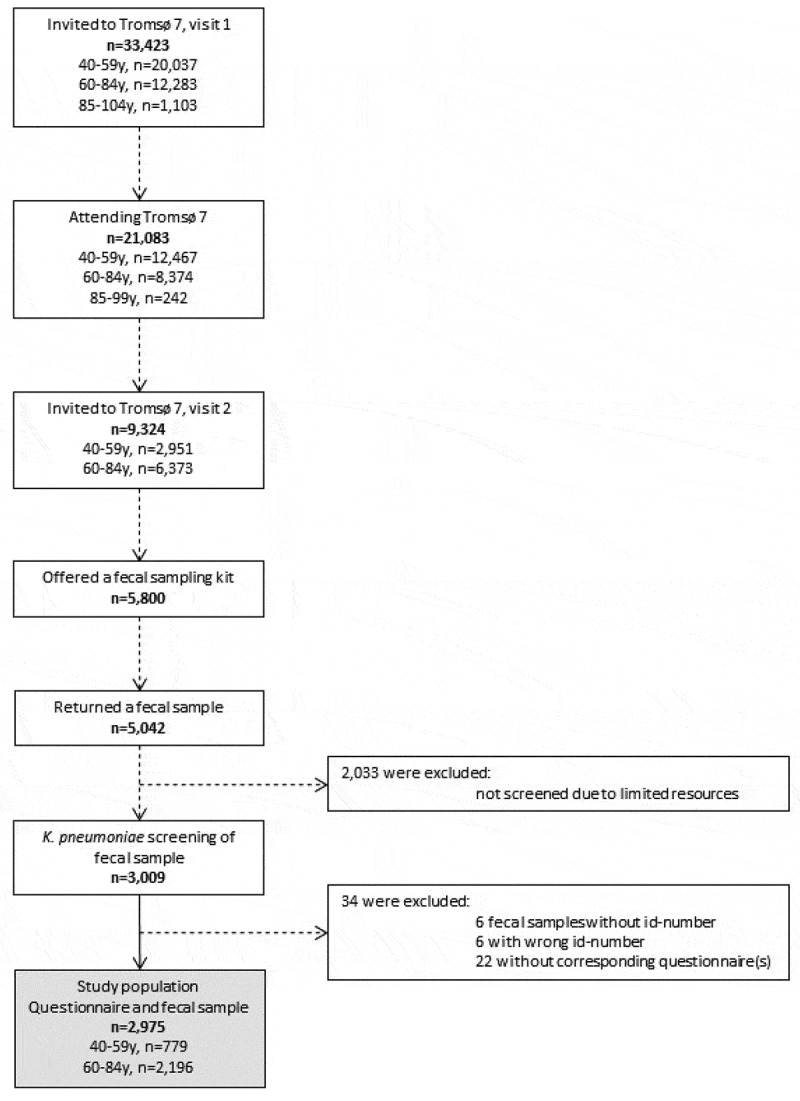
Flow diagram of study population

The Tromsø Study is a population-based study with repeated cross-sectional health surveys in the municipality of Tromsø, Norway. Tromsø is considered as representative of a Northern European, urban population.^43^ The seventh survey of the Tromsø Study (Tromsø 7, https://uit.no/research/tromsostudy) was conducted from March 2015 to October 2016 and included two clinical visits. Unique national identity numbers from the official population-registry were used to invite all citizens >40 y (n = 33,423) (Figure 4). Sixty-five percent (n = 21,083, 11,074 women) attended the first clinical visit in the study. A total of 9,324 participants attending the first visit were invited for a second visit. These represent a random selection of 20% in age-group 40–59 y and 50% in age-group 60–84 y of the initially invited participants (n = 33,423). To enhance the proportion of participants in earlier Tromsø Studies, 3,154 participants aged 40–84 y who had attended clinical examinations in Tromsø 6 were also invited. From March 2015 to March 2016, 5,800 of the 9,324 participants invited for the second visit were consecutively offered a fecal self-sampling kit. In total, 87% (n = 5,042) returned a fecal sample either at the second visit, or by mail to the laboratory. Participants collected fecal material using nylon-flocked ESwab 490CE.A (Copan, Brescia, Italy). The first 3,009 of the 5,042 collected fecal samples were consecutively screened for the presence of Kp via selective culture. Due to resource limitations, the remaining 2,033 samples were not screened. All participants completed two self-administered structured questionnaires on socio-demographics, smoking, alcohol use, hospitalization, chronic diseases, and travel abroad. After excluding 12 participants with wrong or missing sample identification number and 22 with missing questionnaires, our final study population consisted of 2,975 participants.

To assess the participants’ drug use during the preceding 12 months, data from Tromsø 7 were linked to the Norwegian Prescription Database (NorPD, http://www.norpd.no/).^44^ NorPD contains detailed information at the individual level on all dispensed prescription-drugs at all pharmacies in Norway. We defined drugs dispensed as drugs used and included the following groups in the Anatomical Therapeutic Chemical (ATC) classification system: A02 (acid-related disorders), A10 (diabetes), H03A (thyroid hormones), J01, A07AA09, P01AB01 (antibacterials for systemic use), and M01 (anti-inflammatory and anti-rheumatic drugs).

### *Klebsiella pneumoniae* isolation

Upon arrival in the laboratory, 200 µl 85% glycerol was added to each ESwab tube and the samples were stored at −80°C. From the thawed media, 100 µl were plated onto Simmons citrate agar with inositol (SCAI) (both Sigma-Aldrich, Darmstadt, Germany) and incubated for 48 h at 37°C.^42,45^ Large, yellow, glossy colonies suspected of being *Klebsiella* spp. were identified using mass spectrometry (MALDI-TOF, Bruker Daltonics, Bremen, Germany). The first colony identified as either *K. pneumoniae* or *K. variicola* from each sample was kept and further analyzed. All samples were plated on cysteine lactose electrolyte deficient agar (MAST Group, Bootle, UK) to assess growth of fecal flora and validity of the samples.

### Antimicrobial susceptibility testing

Susceptibility testing was performed according to the EUCAST disk diffusion method and interpreted using the EUCAST 2021 breakpoint table (https://eucast.org/).^46^

### Genomic sequencing and bioinformatic analysis

DNA was extracted with the MagNA Pure 96 system (Roche Applied Science, Mannheim, Germany) and sequencing libraries were prepared according to the Nextera Flex sample preparation protocol (Illumina, San Diego, CA, USA). Sequencing was performed on the Illumina MiSeq platform to generate 300 bp paired-end reads. All reads were trimmed with TrimGalore v0.6.4 and assembled with Unicycler v0.4.8 including SPAdes v3.13.0.^47–49^ Kleborate v2.0.0 was used to determine sequence type (ST), species identification, and acquired genes encoding virulence or antibiotic resistance.^50,51^ Kaptive was used to identify capsule biosynthesis loci (KL), and LPS (O) antigen loci.^52,53^ Plasmid replicons were identified with PlasmidFinder v2020-07-13 using Abricate v0.9.9 (https://github.com/tseemann/abricate).^54,55^ Novel STs, *bla*_SHV_, *bla*_LEN_, *bla*_OKP_, and virulence alleles were assigned by the curators of the Institut Pasteur multilocus sequence type (MLST) and core genome MLST databases (https://bigsdb.pasteur.fr/klebsiella). To verify the absence of *bla*_SHV_/*bla*_LEN_, observed in two genomes, the raw reads were inspected with SRST2 v0.2.0.^56^

### Phylogenetic analysis

To assess the phylogenetic relatedness, a core genome alignment of the 484 genomes against the *K. pneumoniae* ST23 NTUH-K2044 reference chromosome (GenBank accession: NC_012731.1) was generated using the RedDog pipeline v1beta.11 (https://github.com/katholt/RedDog), and inferred as described previously.^57,58^ To identify the number of single-nucleotide polymorphisms (SNPs) between any two genomes from the resulting alignment, snp-dists v0.7.0 (https://github.com/tseemann/snp-dists/) was used.

### Definition of hypervirulent Kp

Hypervirulent Kp were defined here according to Huynh *et al*., as isolates harboring at least one of the genes *rmpA* and *rmpA2*, and/or at least one complete gene cluster among *iucABCD-iutA* (aerobactin) and *iroBCDN* (salmochelin).^19^

### Statistical analysis

The primary analysis was a multivariable logistic regression model with outcome variable being culture-confirmed Kp gastrointestinal carriage using SPSS v26.0 (SPSS, Inc., Chicago, IL, USA). *A priori* known or assumed variables associated with Kp carriage were selected with the help of a directed acyclic graph constructed using DAGitty v3.0 (Suppl. Figure 1).^59,60^ All explanatory variables were kept in the fully adjusted model, independent of *p*-values. The strength of the associations was examined by calculating adjusted odds ratios (AORs) with 95% confidence intervals (CIs). Two-sided *p*-values <0.05 were considered statistically significant. Phylogroup subanalyses were performed using *χ*^2^ test. We used R v4.0.0 (Foundation for Statistical Computing, Vienna, Austria) to create a proportional Venn diagram for statistically significant drug groups associated with Kp carriage. STATA v16.1 (StataCorp LLC, Texas, USA) was used to analyze the cumulative change in proportion of Kp carriers associated with antibiotic use in the past 1–12 months.

## Supplementary Material

Supplemental Material

## Data Availability

Bacterial genome data (raw Illumina reads) are publicly available in NCBI under BioProject PRJEB42350. This study is based on data owned by a third party (The Tromsø Study, Department of Community Medicine, UiT The Arctic University of Norway). Confidentiality requirements according to Norwegian law prevent sharing of individual patient-level data in public repositories. Application of legal basis and exemption from professional secrecy requirements for the use of personal health data in research may be sent to a regional committee for medical and health research ethics (https://rekportalen.no/). The authors gained access to the data through the Tromsø Study’s application process. Guidelines on how to access the data are available at the website https://uit.no/research/tromsostudy. All enquiries about the Tromsø Study should be sent by e-mail to tromsous@ism.uit.no. All the questionnaire variables are published in the NESSTAR program system, and results can be viewed online: http://tromsoundersokelsen.uit.no/tromso/.
