# Supplementary material for: Gastrointestinal carriage of Klebsiella pneumoniae in a general adult population: a cross-sectional study of risk factors and bacterial genomic diversity
Source: Gut Microbes. 2021 Jun 28;13(1):1939599. doi: 10.1080/19490976.2021.1939599 (PMC8244762; doi:10.1080/19490976.2021.1939599)
Supplement: Supplemental Material [file KGMI_A_1939599_SM6572.zip › supplementary/20210510_Supplementary_material_Kp T7_Gut Microbes.docx]

**Supplementary material**

**Gastrointestinal carriage of *Klebsiella pneumoniae* in a general population: a cross-sectional study of risk factors and bacterial genomic diversity**

**Authors:** Niclas Raffelsberger (ORCID 0000-0002-9463-8915)^a,b^* Marit Andrea Klokkhammer Hetland (ORCID 0000-0003-4247-8304)^c,d^, Kristian Svendsen (ORCID 0000-0003-3481-3539)^e^, Lars Småbrekke (ORCID 0000-0001-6475-3368)^e^, Iren Høyland Löhr (ORCID 0000-0002-8847-0044)^c^, Lotte Leonore Eivindsdatter Andreassen^a^, Sylvain Brisse (ORCID 0000-0002-2516-2108)^f^, Kathryn E. Holt (ORCID 0000-0003-3949-2471)^g,h^, Arnfinn Sundsfjord (ORCID 0000-0002-3728-2270)^b,i^, Ørjan Samuelsen (ORCID 0000-0002-5525-2614)^e,i⁑^, Kirsten Gravningen (ORCID 0000-0002-8194-3208)^a,j⁑^

^⁑^Contributed equally

*^a^Department of Microbiology and Infection Control, University Hospital of North Norway, Tromsø, Norway*

*^b^Department of Medical Biology, Faculty of Health Sciences, UiT The Arctic University of Norway, Tromsø, Norway*

*^c^Department of Medical Microbiology, Stavanger University Hospital, Stavanger, Norway*

*^d^Department of Biological Sciences, Faculty of Mathematics and Natural Sciences, University of Bergen, Bergen, Norway*

*^e^Department of Pharmacy, Faculty of Health Sciences, UiT The Arctic University of Norway, Tromsø, Norway*

*^f^Institut Pasteur, Biodiversity and Epidemiology of Bacterial Pathogens, Paris, France*

*^g^Department of Infectious Diseases, Central Clinical School, Monash University, Melbourne, Victoria 3004, Australia*

*^h^London School of Hygiene & Tropical Medicine, London WC1E 7HT, UK*

*^i^Norwegian National Advisory Unit on Detection of Antimicrobial Resistance, Department of Microbiology and Infection Control, University Hospital of North Norway, Tromsø, Norway*

*^j^Department of Infection Control and Preparedness, Norwegian Institute of Public Health, Oslo, Norway*

*Corresponding author: Niclas Raffelsberger, Correspondence address: Department of Microbiology and Infection Control, University Hospital of North Norway, N-9038 Tromsø, Norway. E-mail: [niclas.raffelsberger@unn.no](mailto:niclas.raffelsberger@unn.no)

**Table of contents**

Supplementary Table 1. Characteristics for the study population, 2,975 participants in Tromsø 7

Supplementary Table 2. Cross-table analysis of the three most prevalent *Klebsiella* species *K. pneumoniae sensu stricto* (Kp1), *K. quasipneumoniae* subsp. *quasipneumoniae* (Kp2), and *K. variicola* subsp. *variicola* (Kp3) vs. the statistically significant variables analysed in the multivariable model (Table 1).

Supplementary Table 3. Genome characteristics of 484 *K. pneumoniae* species complex isolates (provided as Excel-file)

Supplementary Table 4. SNP matrix of ST35 isolates

Supplementary Table 5. SNP matrix of ST25 isolates

Supplementary Table 6. SNP distances within sequence types with more or equal than five isolates

Supplementary Figure 1. Directed acyclic graph (DAG)

Supplementary Figure 2. Multilocus sequence type (MLST) diversity

Supplementary Figure 3. Prevalence of phenotypic antimicrobial non-susceptibility

Supplementary Figure 4. SHV β-lactamase diversity among *K. pneumoniae sensu stricto* isolates

Supplementary Figure 5. LEN β-lactamase diversity among *K. variicola* subsp. *variicola* isolates

Supplementary Figure 6. OKP-A β-lactamase diversity among *K. quasipneumoniae* subsp. *quasipneumoniae* isolates

Supplementary Figure 7. OKP-B β-lactamase diversity among *K. quasipneumoniae* subsp. *similipneumoniae* isolates

Supplementary Figure 8: Capsule locus (KL) type diversity

Supplementary Figure 9. LPS (O) type diversity

**Supplementary Table 1.** Characteristics for the study population, 2,975 participants in Tromsø 7

| **Characteristics** | **N** | **%** |
| --- | --- | --- |
| **Sex** |  |  |
| Men | 1,360 | 45.7 |
| Women | 1,615 | 54.3 |
| **Age** (years) |  |  |
| 40-49 | 344 | 11.6 |
| 50-59 | 435 | 14.6 |
| 60-69 | 1,286 | 43.2 |
| 70-84 | 910 | 30.6 |
| **Living with a spouse/partner** |  |  |
| No | 676 | 24.1 |
| Yes | 2,124 | 75.9 |
| **Education level** |  |  |
| Low | 1,745 | 60.0 |
| High^a^ | 1,163 | 40.0 |
| **Household income** |  |  |
| Low | 1,250 | 44.6 |
| High^b^ | 1,553 | 55.4 |
| **Current daily smoking** |  |  |
| No | 2,598 | 88.1 |
| Yes | 351 | 11.9 |
| **Alcohol consumption frequency** |  |  |
| Never to ≤monthly | 998 | 33.7 |
| 2-4/month or 2-3/week | 1,765 | 59.6 |
| ≥4/week | 198 | 6.7 |
| **Hospitalization last 12 months** |  |  |
| No | 2,588 | 88.0 |
| Yes | 353 | 12.0 |
| **Diabetes mellitus^c^** |  |  |
| No | 2,674 | 94.0 |
| Yes | 170 | 6.0 |
| **Crohn`s disease/ulcerative colitis** |  |  |
| No | 2,831 | 97.9 |
| Yes | 60 | 2.1 |
| **Travel abroad past 12 months^d^** |  |  |
| No | 1,267 | 42.6 |
| Greece and Asia | 502 | 16.9 |
| All other countries | 1,171 | 39.4 |

^a^ >College/university degree

^b^ ≥551,000 NOK (€ 53,767/year as per January 2021)

^c^ 20 participants who answered “Yes, previously” were excluded

^d^ Travelled outside the Nordic countries >1 week duration in the past 12 months.

**Supplementary Table 2**. Cross-table analysis of the three most prevalent *Klebsiella* species *K. pneumoniae sensu stricto* (Kp1), *K. quasipneumoniae* subsp. *quasipneumoniae* (Kp2), and *K. variicola* subsp. *variicola* (Kp3) vs. the statistically significant variables analysed in the multivariable model (Table 1).

|  | **Kp1 (N*****=2794)** | |  | **Kp2 (N*****=2522)** | |  | **Kp3 (N*****=2625)** | |  |
| --- | --- | --- | --- | --- | --- | --- | --- | --- | --- |
|  | **% (no.)** | | | | | | | | |
|  | **positive (n=303)** | **negative (n=2491)** | **p-value**^a^ | **positive (n=31)** | **negative (n=2491)** | **p-value**^a^ | **positive (n=134)** | **negative (n=2491)** | **p-value**^a^ |
| **Age (years)** |  |  | 0.042 |  |  | 0.004 |  |  | 0.392 |
| 40-49 | 6.4 (21) | 93.6 (307) |  | 0.3 (1) | 99.7 (307) |  | 4.4 (14) | 95.6 (307) |  |
| 50-59 | 10.7 (44) | 89.3 (368) |  | 2.4 (9) | 97.6 (368) |  | 3.7 (14) | 96.3 (368) |  |
| 60-69 | 11.9 (144) | 88.1 (1,064) |  | 0.6 (6) | 99.4 (1,064) |  | 5.8 (65) | 94.2 (1,064) |  |
| 70-84 | 11.1 (94) | 88.9 (752) |  | 2.0 (15) | 98.0 (752) |  | 5.2 (41) | 94.8 (752) |  |
| **Travel abroad past 12 months^b^** |  |  | 0.015 |  |  | 0.838 |  |  | 0.543 |
| No | 9.8 (116) | 90.2 (1,071) |  | 1.2 (13) | 98.8 (1,071) |  | 5.2 (59) | 94.8 (1,071) |  |
| Greece or Asia | 14.5 (68) | 85.5 (400) |  | 1.5 (6) | 98.5 (400) |  | 6.1 (26) | 93.9 (400) |  |
| All other countries | 10.2 (113) | 89.8 (992) |  | 1.1 (11) | 98.9 (992) |  | 4.7 (49) | 95.3 (992) |  |
| **Crohn’s disease/ulcerative colitis** |  |  | 0.025 |  |  | 0.469 |  |  | 0.084 |
| No | 10.7 (286) | 89.3 (2,379) |  | 1.2 (29) | 98.8 (2,379) |  | 4.9 (123) | 95.1 (2,379) |  |
| Yes | 20.4 (11) | 79.6 (43) |  | 0.0 (0) | 100 (43) |  | 10.4 (5) | 89.6 (43) |  |
| **Proton pump inhibitors last 6 m^c^** |  |  | 0.006 |  |  | 0.031 |  |  | 0.003 |
| No | 10.3 (255) | 89.7 (2,228) |  | 1.1 (24) | 98.9 (2,228) |  | 4.7 (109) | 95.3 (2,228) |  |
| Yes | 15.4 (48) | 84.6 (263) |  | 2.6 (7) | 97.4 (263) |  | 8.7 (25) | 91.3 (263) |  |
| **NSAIDs last 6 months^d^** |  |  | 0.326 |  |  | 0.015 |  |  | 0.112 |
| No | 10.6 (255) | 89.4 (2,148) |  | 1.0 (22) | 99.0 (2,148) |  | 4.8 (109) | 95.2 (2,148) |  |
| Yes | 12.3 (48) | 87.7 (343) |  | 2.6 (9) | 97.4 (343) |  | 6.8 (25) | 93.2 (343) |  |
| **Antibiotic systemic use last 1 m^e^** |  |  | 0.002 |  |  | 0.317 |  |  | 0.024 |
| No | 10.5 (283) | 89.5 (2,413) |  | 1.3 (31) | 98.7 (2,413) |  | 4.9 (125) | 95.1 (2,413) |  |
| Yes | 20.4 (20) | 79.6 (78) |  | 0.0 (0) | 100 (87) |  | 10.3 (9) | 89.7 (78) |  |

*N, denominator; m, months; NSAIDs, nonsteroidal anti-inflammatory drugs; drug use according to the Norwegian Prescription Database.

^a^ Determined using the Chi-square test.

^b^ Travelled outside the Nordic countries >1 week duration in the past 12 months.

^c^ A02BC, drugs used for peptic ulcer and gastro-oesophageal reflux disease.

^d^ M01, anti-inflammatory and anti-rheumatic products (non-steroids), anti-inflammatory/anti-rheumatic agents in combination and specific anti-rheumatic agents.

**^e^** J01, A07AA09, P01AB01, antibacterials for systemic use, intestinal antiinfectives and nitroimidazole derivates used as antiprotozoals (metronidazole).

**Supplementary Table 3. ->** provided as Excel table

**Supplementary Table 4**. Matrix showing SNP differences among nine *K. pneumoniae sensu stricto* ST35 isolates

|  | **T7-208** | **T7-272** | **T7-276** | **T7-290** | **T7-330** | **T7-358** | **T7-360** | **T7-396** | **T7-479** |
| --- | --- | --- | --- | --- | --- | --- | --- | --- | --- |
| **T7-208** |  | 311 | 310 | 310 | 310 | 312 | 310 | 309 | 272 |
| **T7-272** | 311 |  | 1 | 1 | 4 | 1 | 0 | 2 | 309 |
| **T7-276** | 310 | 1 |  | 0 | 1 | 2 | 0 | 1 | 308 |
| **T7-290** | 310 | 1 | 0 |  | 0 | 2 | 0 | 1 | 308 |
| **T7-330** | 310 | 4 | 1 | 0 |  | 5 | 1 | 1 | 311 |
| **T7-358** | 312 | 1 | 2 | 2 | 5 |  | 0 | 3 | 310 |
| **T7-360** | 310 | 0 | 0 | 0 | 1 | 0 |  | 1 | 308 |
| **T7-396** | 309 | 2 | 1 | 1 | 1 | 3 | 1 |  | 309 |
| **T7-479** | 272 | 309 | 308 | 308 | 311 | 310 | 308 | 309 |  |

**Supplementary Table 5**. Matrix showing SNP differences among five *K. pneumoniae sensu stricto* ST25 isolates

|  | **T7-067** | **T7-127** | **T7-161** | **T7-308** | **T7-451** |
| --- | --- | --- | --- | --- | --- |
| **T7-067** |  | 30 | 3,066 | 29 | 29 |
| **T7-127** | 30 |  | 3,045 | 7 | 7 |
| **T7-161** | 3,066 | 3,045 |  | 3,044 | 3,044 |
| **T7-308** | 29 | 7 | 3,044 |  | 8 |
| **T7-451** | 29 | 7 | 3,044 | 8 |  |

**Supplementary Table 6.** SNP ranges within sequence types (ST) with more or equal than five *K. pneumoniae sensu stricto* isolates

| **Sequence Type** | **No. of genomes** | **SNP range** |
| --- | --- | --- |
| **ST10** | 7 | 70-208 |
| **ST14** | 7 | 22-3,897 |
| **ST20** | 15 | 119-8,278 |
| **ST25** | 5 | 7-3,066 |
| **ST26** | 13 | 39-4,885 |
| **ST35** | 9 | 0-312 |
| **ST37** | 9 | 757-16,055 |
| **ST45** | 8 | 68-3,348 |
| **ST253** | 5 | 107-6,508 |
| **ST461** | 8 | 24-4,263 |
| **ST641** | 7 | 122-162 |
| **ST643** | 5 | 55-96 |
| **ST681** | 7 | 60-200 |
| **ST1423** | 5 | 115-396 |
| **ST1562** | 6 | 39-11,993 |
| **ST2004** | 5 | 13-23 |
| **ST2386** | 9 | 41-78 |

**
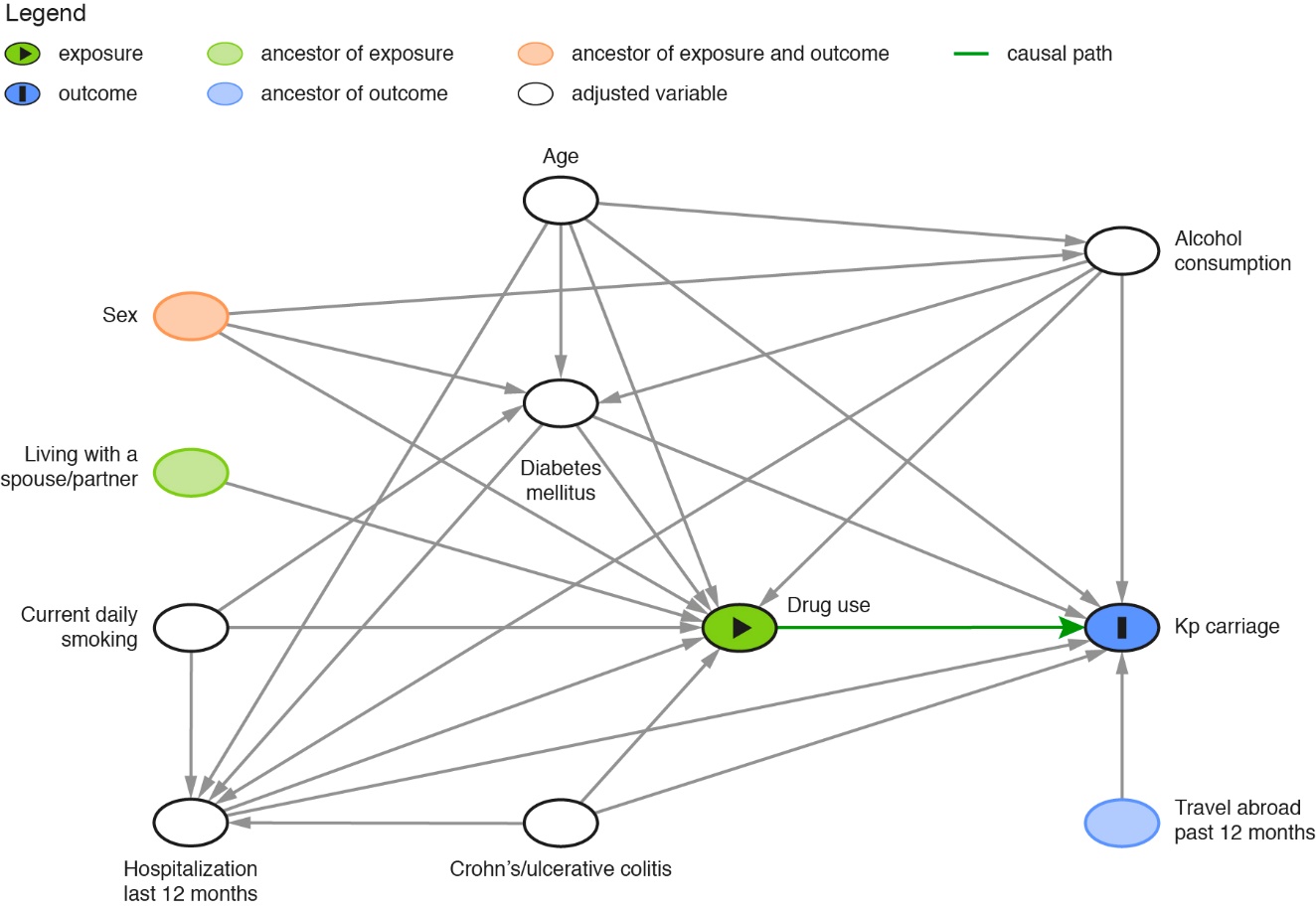
**

**Supplementary Figure 1**. Directed acyclic graph (DAG) illustrating causal relationships between drug use (exposure), *K. pneumoniae* (Kp) faecal carriage (outcome) and relevant covariates. DAG was used for selection of the multivariable logistic regression model. White variables are those adjusted for. Even if sex is an ancestor of outcome, there is no biasing path implying that sex should not be included in the model. The drug use variables include proton pump inhibitors, non-steroidal anti-inflammatory drugs, metformin and thyroid hormones used the last six months, and antibacterials for systemic use the last one month.


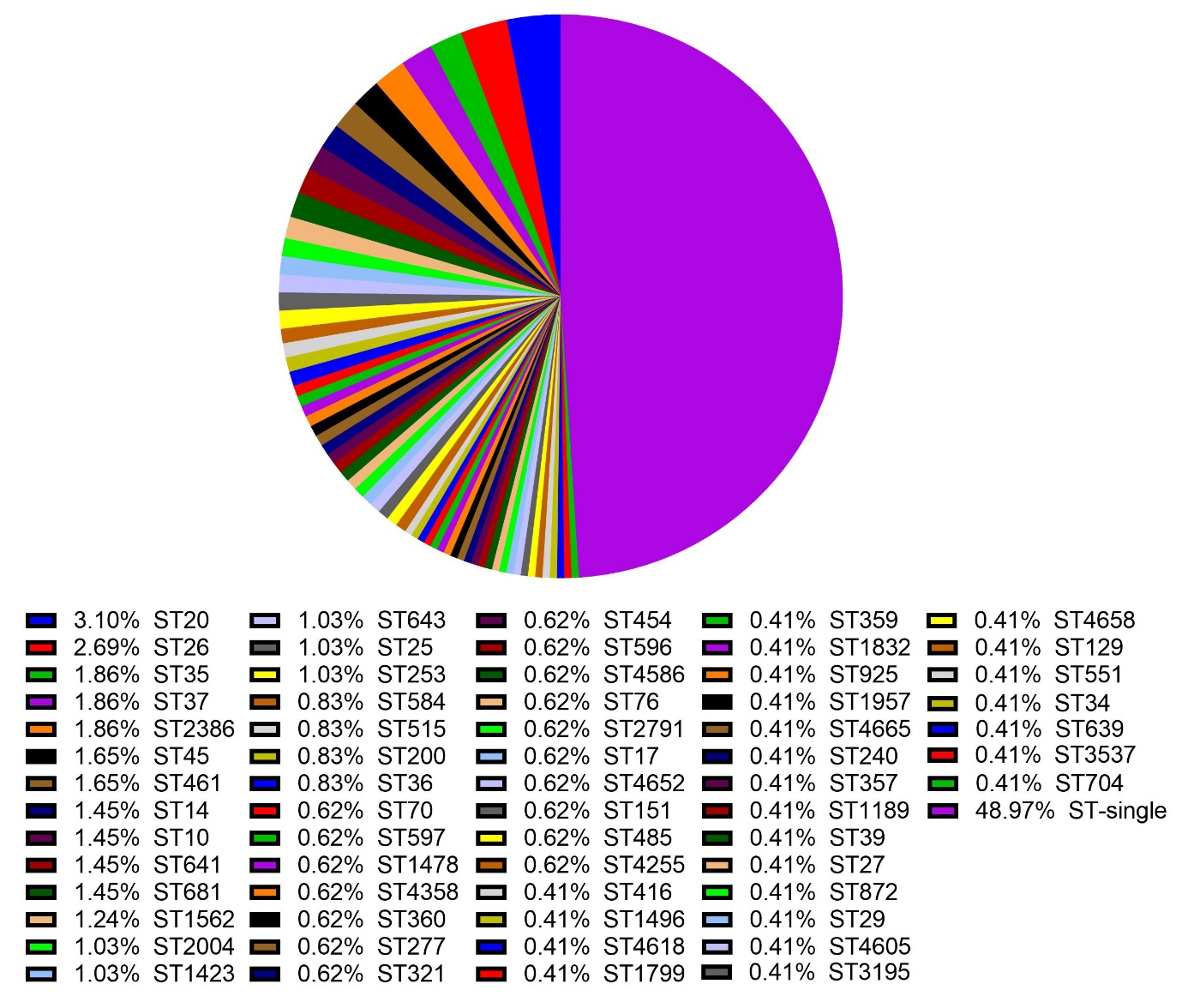
**Supplementary Figure 2**. Multilocus sequence type diversity in 484 *K. pneumoniae* species complex isolates including 300 different sequence types (STs). ST and proportion (%) is indicated in the colour legend. Unique STs represented by one isolate is grouped in the ST-single (large violet) area. In total, 32% (96 of 300) of STs identified were novel.


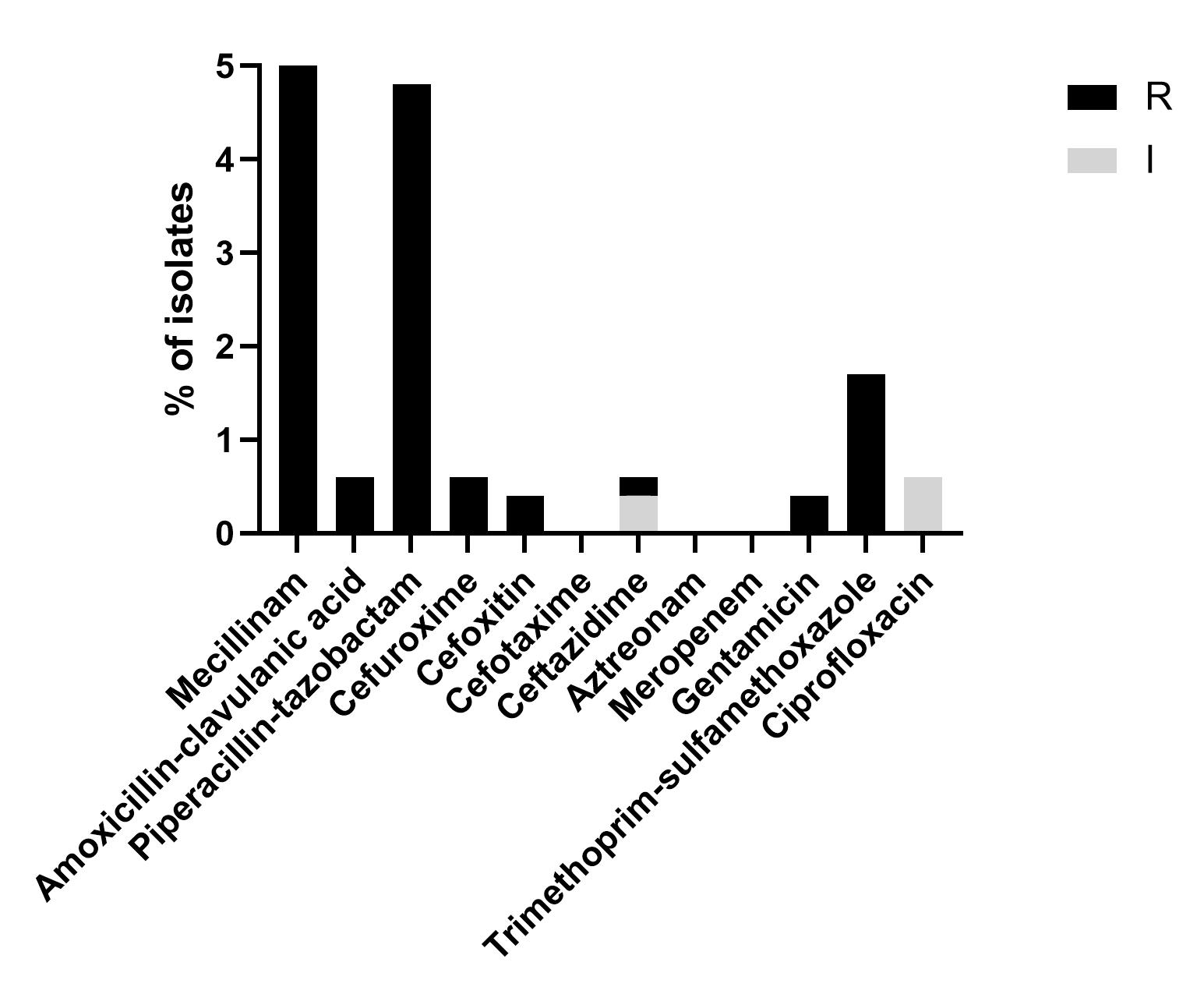


**Supplementary Figure 3**. Prevalence of phenotypic antibiotic resistance (R) and susceptible, increased exposure (I) among 484 *K. pneumoniae* species complex isolates.


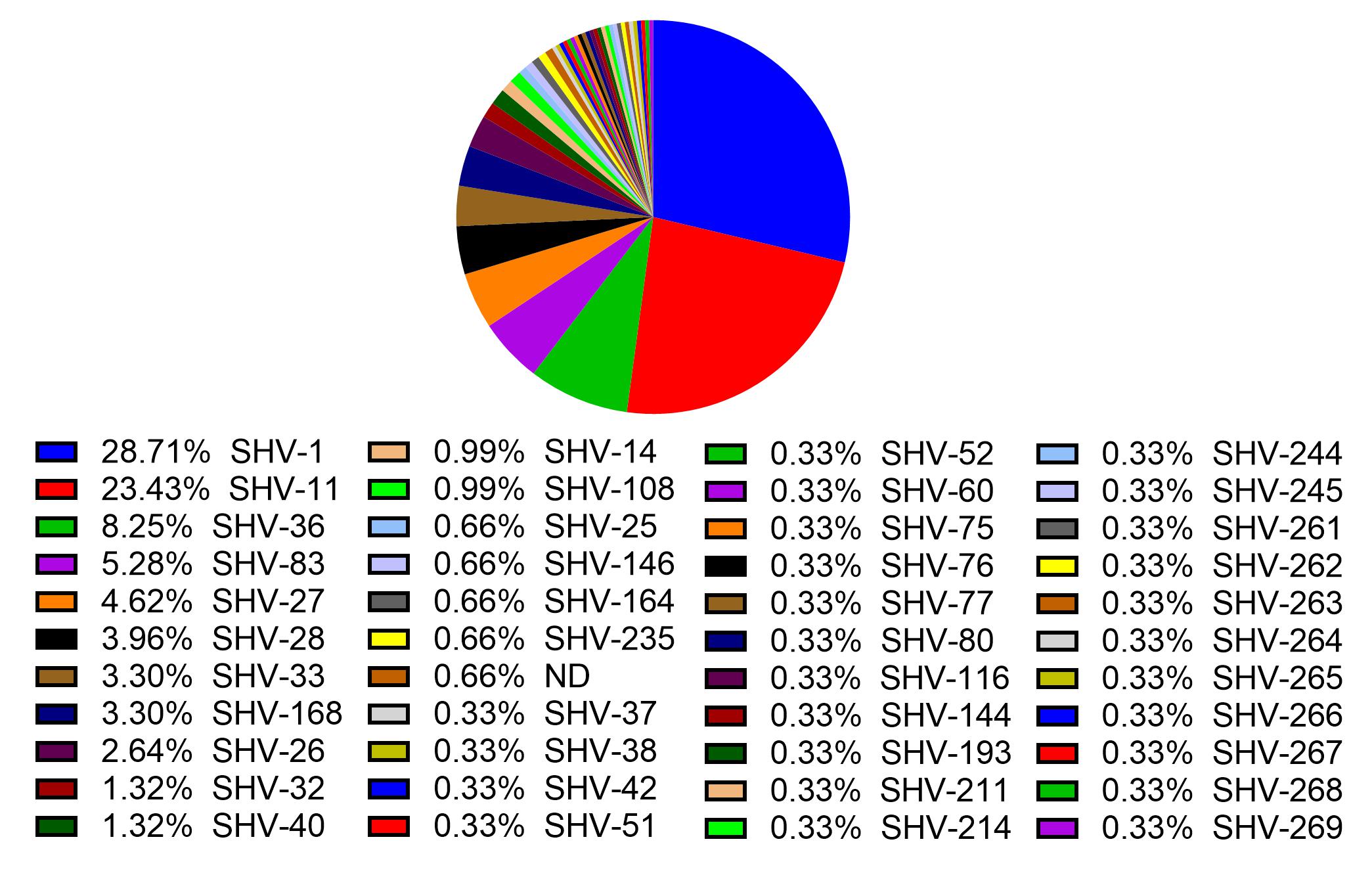


**Supplementary Figure 4.** SHV β-lactamase diversity among *K. pneumoniae sensu stricto* (Kp1) isolates (n=303). SHV-variant and proportion (%) is indicated in the legend. Two isolates with either a deleted *bla*_SHV_ gene or a *bla*_SHV_ gene with a premature stop codon are included and labelled ND.


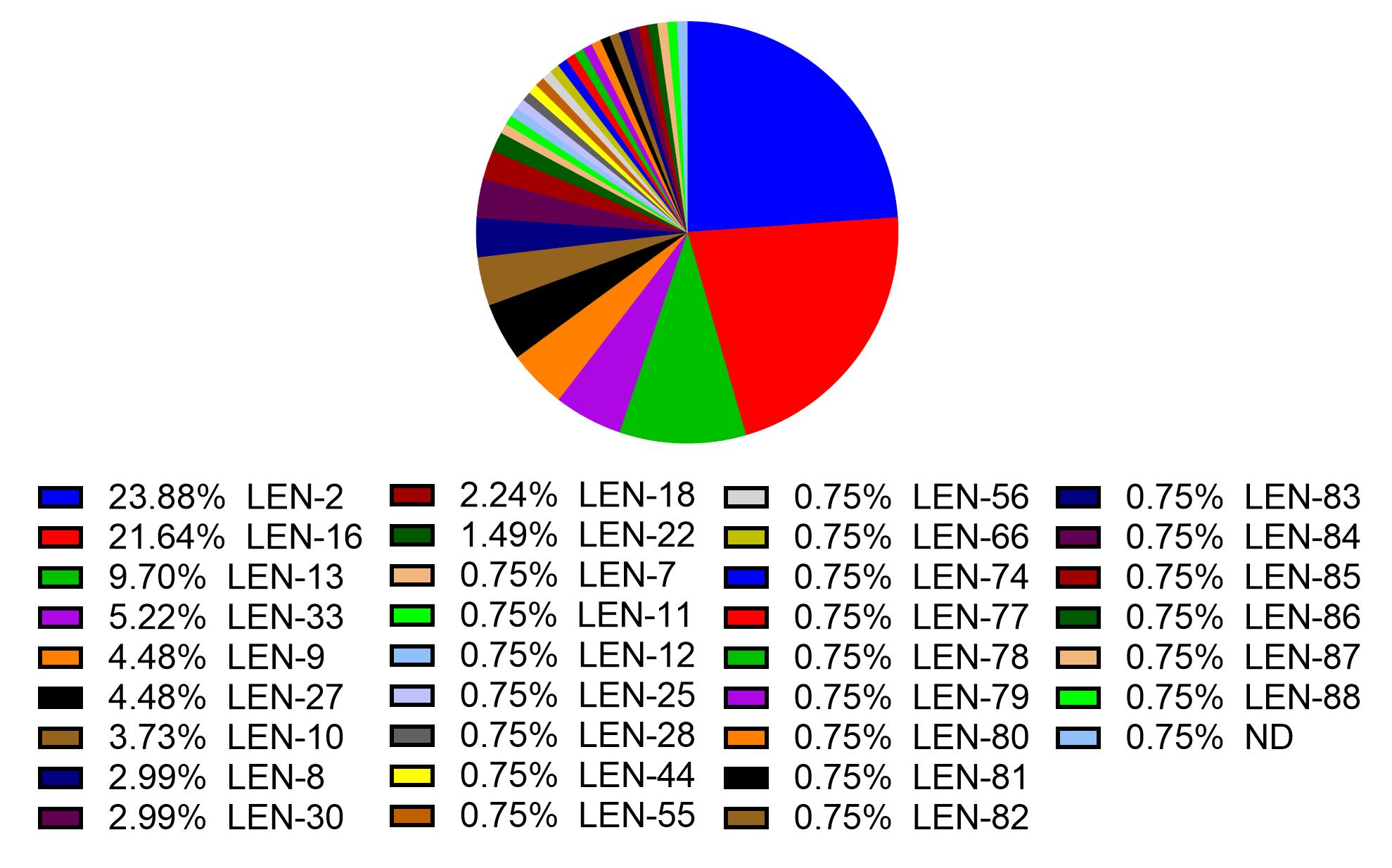


**Supplementary Figure 5**. LEN β-lactamase diversity among *K. variicola* subsp. *variicola* (Kp3) isolates (n=134). LEN-variant and proportion (%) is indicated in the legend. One isolate with a deleted *bla*_LEN_ gene is included and labelled ND.


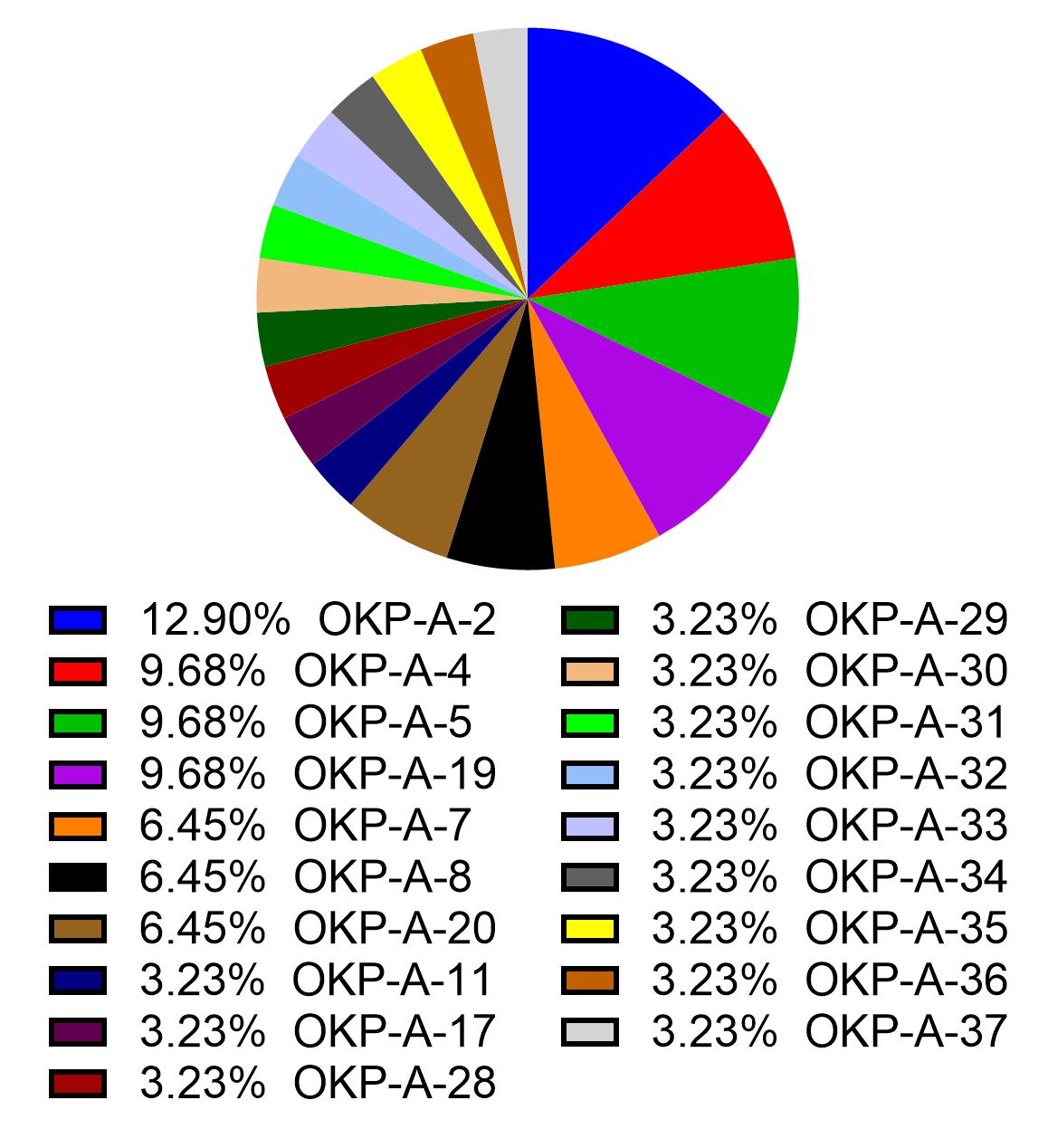


**Supplementary Figure 6**. OKP-A β-lactamase diversity among *K. quasipneumoniae* subsp. *quasipneumoniae* (Kp2) isolates (n=31). OKP-A-variant and proportion (%) is indicated in the legend.


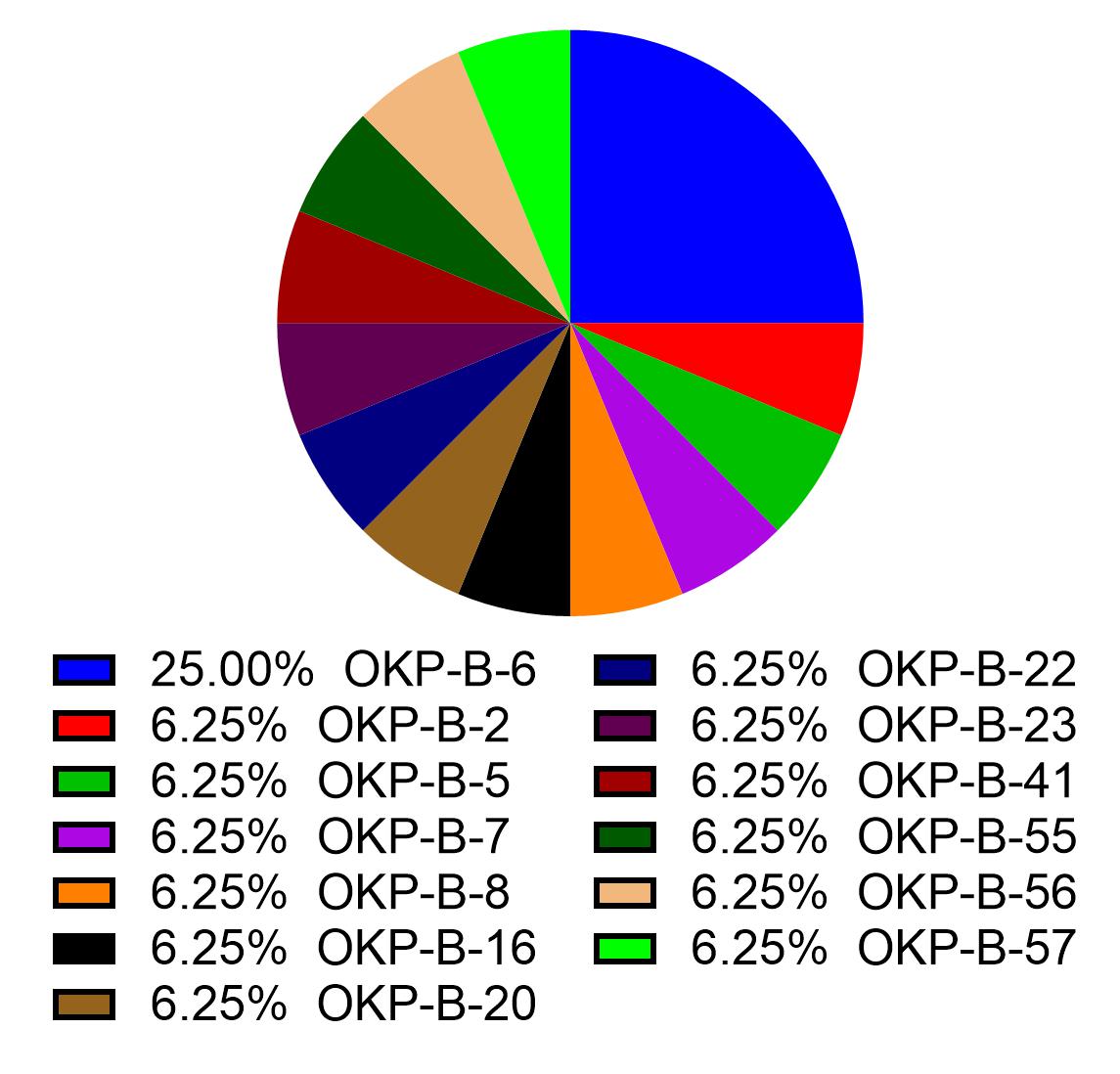


**Supplementary Figure 7**. OKP-B β-lactamase diversity among *K. quasipneumoniae* subsp. *similipneumoniae* (Kp4) isolates (n=16). OKP-B-variant and proportion (%) is indicated in the legend.


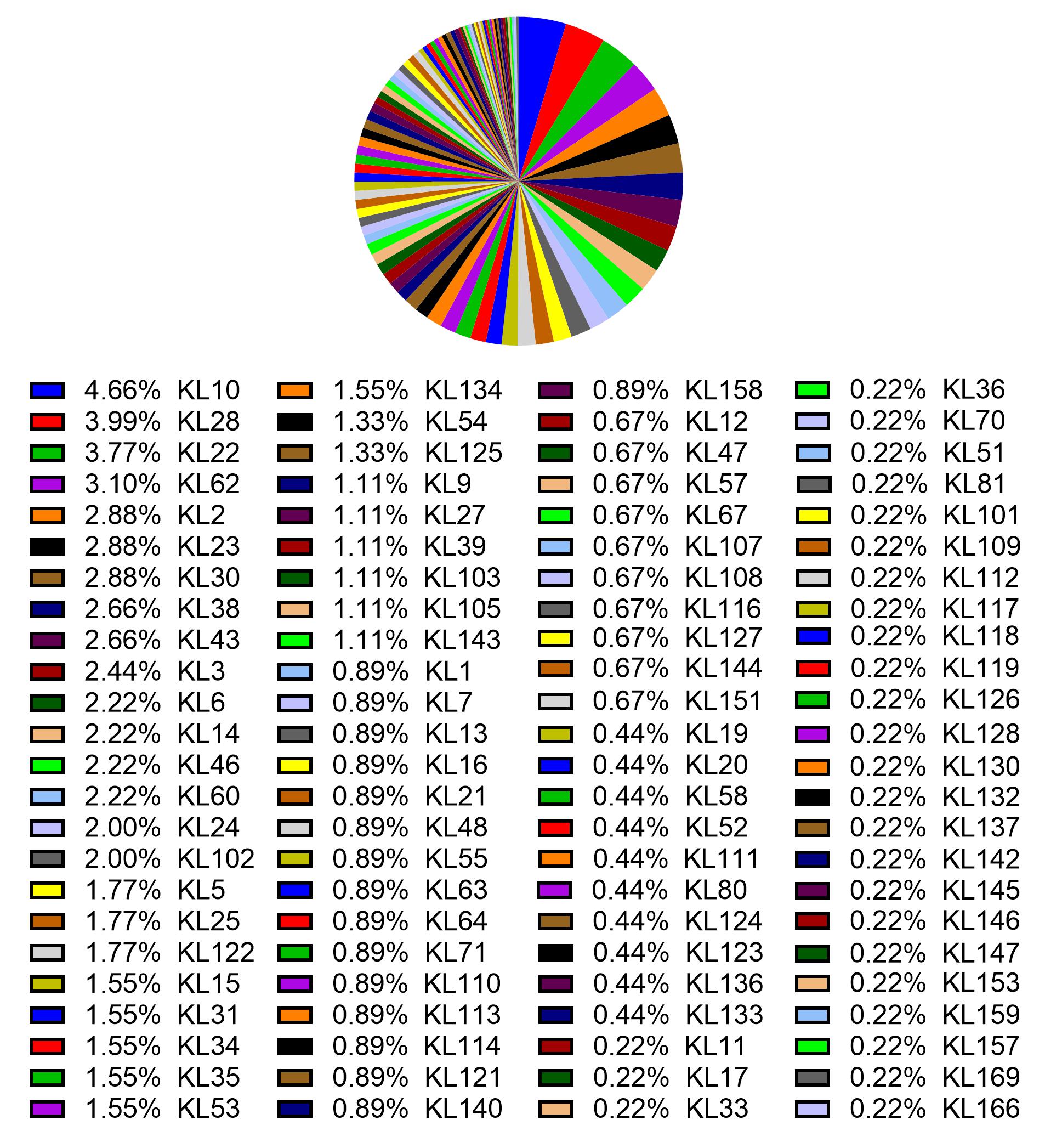


**Supplementary Figure 8**: Capsule locus (KL) type diversity among 451 *K. pneumoniae* species complex isolates with a defined KL type and proportion (%) indicated in the legend.^1^


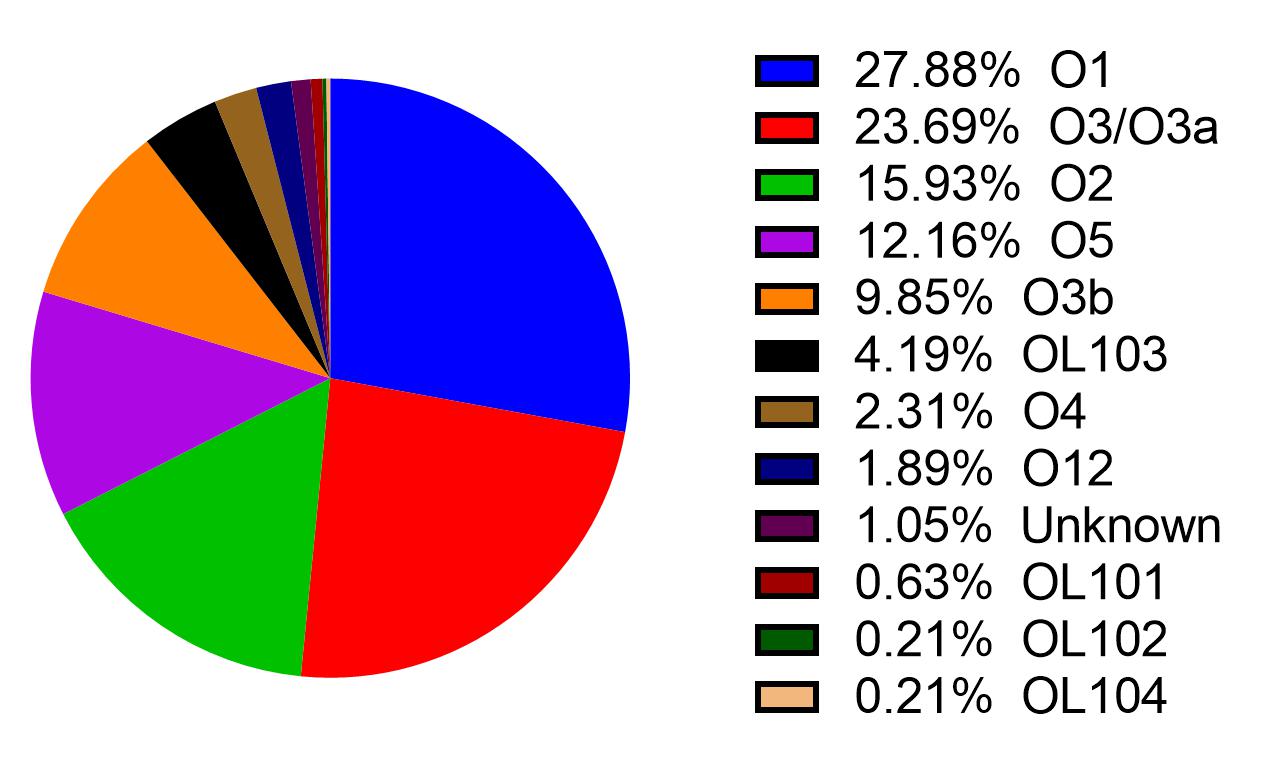


**Supplementary Figure 9**. O type diversity among 477 *K. pneumoniae* species complex isolates with a defined O type and proportion (%) is indicated in the legend. Unknown represents when only one of the two additional genes (*wbbY* and *wbbZ*) can be found, the result is ambiguous and can either be O1 or O2.^1^

**References**

1.Wick RR, Heinz E, Holt KE, Wyres KL. Kaptive Web: User-Friendly Capsule and Lipopolysaccharide Serotype Prediction for *Klebsiella* Genomes. *J Clin Microbiol. 2018 May 25;56(6):e00197-18.*
